# Proteomics-Based Study of Potential Emphysema Biomarkers Reveals Systemic Redox System and Extracellular Matrix Component Dysregulation

**DOI:** 10.3390/diagnostics16060931

**Published:** 2026-03-21

**Authors:** Grgur Salai, Ruđer Novak, Stela Hrkač, Václav Pustka, David Potěšil, Zbyněk Zdráhal, Divo Ljubicic, Lovorka Grgurević

**Affiliations:** 1 Department of Pulmonology, University Hospital Dubrava, 10 000 Zagreb, Croatia; salai.grgur@gmail.com (G.S.); divo.ljubicic@gmail.com (D.L.); 2 Department of Proteomics, Center for Translational and Clinical Research, School of Medicine, University of Zagreb, 10 000 Zagreb, Croatia; rudjer.novak@mef.hr; 3 BIMIS—Biomedical Research Center Šalata, School of Medicine, University of Zagreb, 10 000 Zagreb, Croatia; 4 Department of Clinical Immunology, Allergology and Rheumatology, University Hospital Dubrava, 10 000 Zagreb, Croatia; stela.hrkac@gmail.com; 5 Central European Institute of Technology, Masaryk University, 612 00 Brno, Czech Republic; vaclav.pustka@ceitec.muni.cz (V.P.); david.potesil@ceitec.muni.cz (D.P.); zdrahal@sci.muni.cz (Z.Z.); 6 Department of Internal Medicine, School of Medicine, University of Zagreb, 10 000 Zagreb, Croatia; 7 Department of Anatomy, “Drago Perović”, School of Medicine, University of Zagreb, 10 000 Zagreb, Croatia

**Keywords:** emphysema, COPD, ME1, BMP1, anoikis, extracellular matrix, redox homeostasis

## Abstract

**Objective:** Emphysema is an important chronic obstructive pulmonary disease (COPD) phenotype characterized by the destruction of air spaces distal to the terminal bronchiole. Aiming to detect potential emphysema biomarkers and to assess the systemic effects of emphysema in blood plasma, we conducted a small cross-sectional shotgun proteomics study. **Methods:** This study included N = 40 participants divided into four subgroups (N = 10 per group): patients with emphysema and COPD (CE), patients with COPD but without emphysema (CN), healthy smokers (HS) and healthy never-smokers (HN). The participants were sampled non-probabilistically to be similar in terms of age, sex and comorbidities. Participants’ blood plasma was analyzed using liquid chromatography–mass spectrometry. Bioinformatic analysis included detection of differentially expressed proteins (DEPs) and overrepresentation analysis (ORA). **Results:** Across all groups, a total of 994 proteins were identified, with NADP-dependent malic enzyme (NADP-ME; encoded by *ME1*) being the only DEP in the CE vs. CN contrast. Proteins such as BMP1, ADAMTSL-2, -4 and IGFBP4, -5, 6 were identified to be upregulated in CE vs. HN. Fibulin-1, -3 and several immunoglobulin components were identified to be downregulated in the CE vs. HN contrast. ORA revealed several enriched processes, including serine-type endopeptidase activity, insulin-like growth factor I and II binding, and signaling receptor binding. **Conclusion:** We propose NADP-ME, an important enzyme of intermediary metabolism and redox homeostasis, as a potential biomarker candidate of emphysema. Notably, NADP-ME is also implicated in anoikis resistance. Additionally, changes in the expression levels of BMP1, ADAMTSL-2 and -4, and fibulin suggest potential major systemic effects of extracellular matrix perturbation. As all data was derived from LC-MS analysis, these findings need to be further evaluated with complementary methods.

## 1. Introduction

Chronic obstructive pulmonary disease (COPD) is a heterogenous condition characterized by chronic respiratory symptoms and abnormalities of the airways and/or alveoli (emphysema) that cause persistent, often progressive airflow obstruction [1]. It is considered that COPD results from gene–environment interactions occurring over the individual’s lifetime that damage the lungs and alter their normal aging process. Cigarette smoking has long been recognized as a key environmental risk factor for COPD development [1].

Emphysema is a condition (and COPD phenotype) that affects the air spaces distal to the terminal bronchiole, marked by abnormal and permanent enlargement of lung air spaces, destruction of air space walls (historically regarded to be without fibrosis, but local fibrosis may coexist) and loss of lung parenchyma elasticity [2,3,4]. At the structural level, emphysema primarily affects the alveolar wall (a delicate composite of epithelial and endothelial cells anchored to a highly specialized extracellular matrix (ECM) rich in elastin), whose integrity is essential for gas exchange and mechanical stability [2,5]. The pathogenesis of emphysema (and COPD) is very complex and incompletely understood [6]. Several pathophysiologic mechanistic axes, which are likely interconnected, in which imbalances occur, have been proposed [5,6]. The most famous is the imbalance of the protease–antiprotease system. Namely, cigarette smoking induces abnormal inflammation, which creates a protease-rich environment, and thus, an imbalance between proteases and antiproteases occurs [5]. Another hypothesis considers oxidant–antioxidant dysregulation, which highlights the fact that exposure to irritants (most notably cigarette smoke) leads to an increase in exogenous oxidants in the lungs. These irritants also lead to the recruitment of inflammatory cells, which are in turn responsible for the production of endogenous oxidants (and elastases), thus aggravating the condition [5]. Additionally, ROS activate the transcription of proinflammatory cytokines via the NF-kB pathway, thus completing the vicious cycle [7]. However, a counterbalance system comprising an elaborate antioxidant network exists, which is of paramount importance for maintaining redox homeostasis [7]. This system comprises non-enzymatic molecules (such as glutathione, vitamins C and E, and taurine) which act as a first line of defense against ROS. Additionally, several enzymes (including superoxide dismutases, peroxiredoxins, thioredoxins and glutaredoxins) act in concert with non-enzymatic antioxidants to maintain cellular redox homeostasis [7]. Reactive oxygen species (ROS) target host macromolecules, which results in cell dysfunction and death [5]. Importantly, several types of programmed cell death have been recognized as an emerging driving mechanism of emphysema pathogenesis [5,8]. Furthermore, elastic fiber injury in the lungs plays a detrimental role in the initiation and progression of emphysema. Specifically, elastic fibers prevent airway collapse and allow passive expiration (elastic recoil). Elastic fibers such as elastin and its microfibrils are known to be impacted by protease degradation and direct oxidation of elastin, which reduces lung elasticity and ultimately leads to impaired gas exchange [5,9]. Additionally, elastin antibodies have been identified, and the role of autoimmunity in emphysema development is currently being explored [10,11].

In clinical practice, emphysema is an important COPD phenotype: specifically, emphysema often leads to secondary pulmonary hypertension and muscle wasting, it is an independent risk factor for lung cancer, and there is evidence that individuals with upper lobe emphysema are at higher risk for rapid lung function decline [1,12,13,14]. Nowadays, emphysema is commonly diagnosed using chest computed tomography (CT) scans, visible as areas of low attenuation, usually without visible walls [3,4]. However, in order to mitigate risks of radiation, identification of emphysema biomarkers is an important research area as the discovery of emphysema biomarkers could potentially lead to a more personalized approach to COPD management. Several blood biomarkers of emphysema, such as increased adiponectin or decreased advanced glycosylation end product-specific receptor variant (sRAGE), have been proposed, but they have not been validated in large cohorts and are not used in clinical practice [15].

We conducted a small cross-sectional shotgun proteomics study which included COPD patients with emphysema (CE), COPD patients without emphysema (CN) (with similar clinical characteristics in terms of airflow limitation, symptomatic burden, inhalation therapy and comorbidities), healthy smokers (HS), and healthy never-smokers (HN), aiming to explore the systemic effects of (smoking-induced) emphysema and to identify its potential biomarkers.

## 2. Materials and Methods

We conducted a small cross-sectional proteomic study aiming to identify potential biomarkers of emphysema and to identify COPD- and smoking-related biomarkers. This study was approved by the University Hospital Dubrava’s Institutional Ethics Committee (approval no. 2022/2908-05; approval date 31 August 2022) and by the School of Medicine, University of Zagreb’s Institutional Ethics Committee (approval no. 251-59-10106-24-111/119, approval date 23 September 2024).

**Figure 1:**
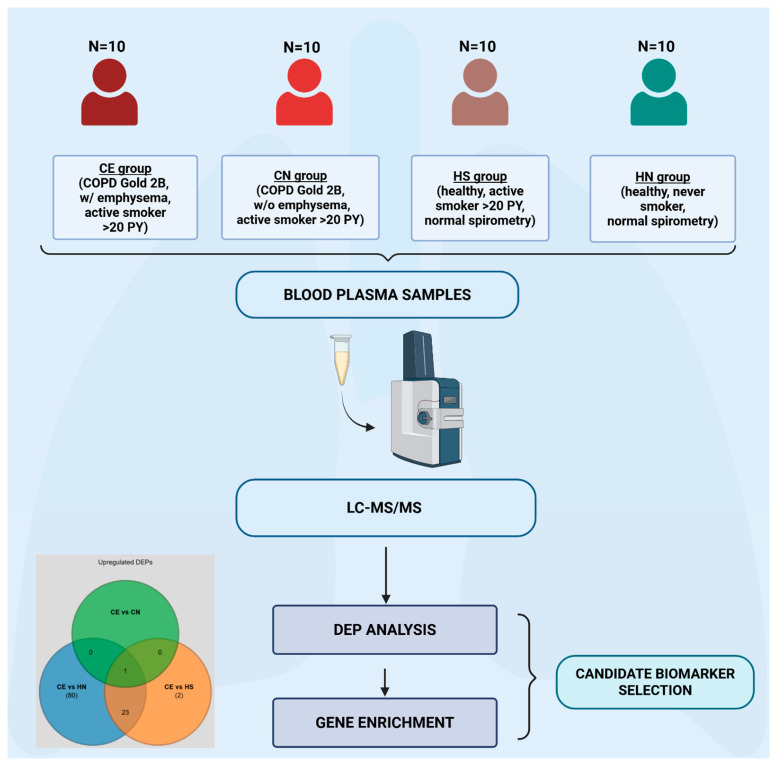
Study outline depicting study groups and methods. The study included four study groups: (1) patients with COPD and emphysema visible on chest CT (CE), (2) patients with COPD without emphysema visible on chest CT (CN), (3) healthy active smokers (>20 pack-years) (HS), and (4) healthy-never smokers (HN). There were N = 10 participants per group. Blood samples were analyzed by LC-MS after which differentially expressed proteins (DEPs) were identified and gene enrichment analysis (i.e., overexpression analysis (ORA)) was performed. Created in BioRender. Hrkač, S. (2026) https://BioRender.com/sv5czjj (accessed 15 March 2026).

This study included a total of 40 participants, divided into four equal age- and gender-matched groups (N = 10 participants per group): (1) patients with COPD and radiologically verified emphysema (with a threshold of -950 HU) on chest computed tomography (CT) (CE group), confirmed by a radiologist; (2) patients with COPD without radiological signs of emphysema on chest CT (CN group), confirmed by a radiologist; (3) healthy smokers (active smokers (>20 pack-years) without respiratory symptoms and normal spirometry) (HS group); and (4) healthy never-smokers (without respiratory symptoms and with normal spirometry) (HN group). A graphic study outline is shown in Figure 1.

Participants were included in the study in a non-probabilistic manner in order to be matched by age, gender, comorbidity and body mass index to ensure group homogeneity among these factors. Patients with COPD were recruited from the Department of Pulmonology’s out-patient clinic, and healthy individuals were recruited from the University Hospital Dubrava’s Polyclinic after they underwent a health-screening examination. After being invited to participate and accepting, all participants in the study signed an informed consent form.

Patients with COPD (CE and CN groups) were GOLD 2B patients as defined by GOLD 2023 [16], active smokers (>20 pack-years), and on dual inhalation therapy (combination of long-acting β_2_ agonist (LABA) and long-acting muscarinic antagonist (LAMA)). Healthy participants were people without respiratory symptoms and with normal spirometry. Exclusion criteria for all participants were: reversible airflow limitation, positive bronchodilator test (either by the GINA criteria or by the ERS/ATS technical standard 2022 [16,17]), concomitant malignancy, autoimmune disease or concomitant asthma, and alpha-1-antitrypsin deficiency. Participants that consumed tobacco products other than classical cigarettes (including e-cigarettes, heat-not-burn tobacco products, vapes, cigars and cigarillos) were also excluded from the study. None of the participants were taking glucocorticoids or other immunosuppressive medications. Exclusion criteria for COPD patients was an acute exacerbation of COPD in the last 6 months. No participant had significant exposure to biomass fuels.

Comorbidities were assessed using the Charlson’s comorbidity index and also a modified Charlson’s comorbidity index in which “chronic pulmonary disease” was excluded. The participants underwent spirometry testing, and all COPD patients had a chest CT no older than 3 months prior to inclusion. Due to ethical concerns of exposing the healthy participants to radiation without clinical indication, they were not required to undergo a chest CT in order to be included in the study. Spirometry with a subsequent bronchodilator test (with 400 mcg of salbutamol) was performed according to the international standards, using Global Lung Initiative (GLI) reference values [17,18,19]. Expiratory airflow limitation was determined using the proposed GOLD criteria with a fixed FEV1/FVC ratio of 0.7 [16].

Participants’ characteristics (displayed in Table 1) were analyzed by employing descriptive statistical methods, using jamovi 2.6.44. Type one error (alpha) was set at 0.05. Data distribution normality was assessed with Shapiro–Wilk’s test. Variables with parametric distribution were analyzed using the one-way analysis of variance (ANOVA) test with the Games–Howell post hoc test, in case of statistical significance. Non-parametric variables were formally assessed with the Kruskal–Wallis test, followed by Dwass–Steel–Critchlow–Flinger (DSCF) pairwise comparisons in the case of a statistically significant result. As expected, participants with COPD (CE and CN) differed from the healthy individuals (HS and HN) in terms of FEV1, FVC and FEV1/FVC values and Charlson’s comorbidity index. When a modified score of Charlson’s comorbidity index (in which we omitted the category for “chronic pulmonary disease”) was employed, there was no statistically significant difference between the groups. The Games–Howell post-hoc test for FEV1 and FVC, as well as the DSCF pairwise comparison for FEV1/FVC, did not reveal statistically significant differences between CE vs. CN and HS vs. HN.

Venipuncture was performed in order to draw blood samples and place them in a vacuette (3.8% sodium citrate tubes), and the samples were centrifuged for 15 min at 4 °C and 3000 *g* to obtain plasma and then stored at −80 °C until further analysis. Before use, samples were thawed and centrifuged at 16,000 *g* for 10 min to remove residual larger debris and the supernatant was used in subsequent experiments. Total protein concentration was determined using the RC DC Lowry protein assay (BioRad, Hercules, CA, USA; #5000122) according to the manufacturer’s instructions. Samples containing 100 μg of protein per participant were transferred to 10 kDa centrifugal filter units for further processing. Briefly, proteins were denatured in 8 M urea, alkylated in 55 mM iodoacetamide (in 8 M urea), and finally digested overnight in 25 mM ammonium bicarbonate with 1 μg of TPCK-treated trypsin (Worthington Industries, Columbus, OH, USA; #11418025001), as described previously [20]. The obtained tryptic peptides were desalted and concentrated using in-house-made Stage Tips mini-columns, as described previously [20,21].

Peptides were extracted for LC-MS using 80% acetonitrile (ACN) in 0.1% formic acid (FA). LC-MS/MS analysis was done using the UltiMate 3000 RSLCnano system (Thermo Fisher Scientific) and the timsTOF HT (Bruker). Before LC separation, peptides were concentrated and desalted online and then separated using an analytical column (EASY-Spray column, 75 μm ID, 250 mm long, 2 μm particles, Thermo Fisher Scientific; #ES902) for 90 min in a gradient of ACN/H_2_O (FA). DIA LC-MS data processing was performed using DIA-NN application (version 2.1.0) [22,23]. Library-free search mode was applied using the custom iRTs_trypsin and UniProtKB-Human databases specificity. Match between runs (MBR) was used across the whole dataset, and the database search results were set to follow the false discovery rate (FDR) thresholds: precursor level, 1% FDR; protein group level, 1% FDR. Fixed algorithm settings were used for the search (MS2 and MS1 accuracy of 15 and 15 ppm, respectively, with scan window 11), and the default protein inference algorithm implemented in DIA-NN was used to construct the list of protein groups utilizing proteotypic peptides (i.e., peptides unique for the given protein within the whole protein database). Two outlier samples per experimental group were identified using cluster analysis and sample-to-sample correlation analysis. Samples showing inconsistent clustering and deviating strongly from their respective experimental groups were excluded from further analysis. Precursors were filtered for those being quantified in 60% of replicates in at least one sample group. Filtered precursor intensities (Precursor.Normalised column from the main DIA-NN report, i.e., raw precursor intensities normalized internally by DIA-NN) were further normalized using loessF function, and normalized precursor intensities were imputed using global quantile (0.001) value; normalized and imputed precursor intensities were used to calculate MaxLFQ protein level intensities using iq R package [24] for relative protein abundance evaluation. Filtered and normalized precursor intensities were used to calculate DIA-TPA protein level intensities useable as absolute protein abundance estimates [25] (MaxLFQ values were used for LIMMA statistical processing).

The MS raw data were deposited at the ProteomeXchange Consortium via the PRIDE partner repository and are available via ProteomeXchange with identifier PXD074107.

For comparisons between study groups (*CE* vs. *CN*, *CE* vs. *HS* and *CE* vs. *HN*), linear models for microarray and RNA data (LIMMA) *t*-test was used with Benjamini & Hochberg adjustment for multiple hypothesis testing [26,27]. Proteins with an adjusted *p*-value < 0.05 and a log_2_-transformed fold change (FC) of log_2_(FC) > 1 or log_2_(FC) < −1 were deemed statistically significant. Data were visualized in the in-house built software Proteo Visualizer 3.0.8 (Cupak, M. Proteo-Visualizer 2026 (CEITEC, Brno, Czech Republic)).

Gene enrichment was performed via the ShinyGO 0.85 platform [28] by employing STRING [28,29]. Overrepresentation analysis (ORA) was performed with an FDR cutoff of 0.05. This was conducted only for the identified DEPs from the CE vs. HN contrast to avoid redundancy (due to the fact that 23 out of 25 (92%) of upregulated DEPs identified in CE vs. HS were also identified as DEPs in CE vs. HN).

Additionally, protein–protein interactions for the DEPs in the CE vs. HN contrast were visualized using STRING (version 12.0) [29]. Cluster analysis was performed within the STRING platform using the Markov cluster algorithm (MCL) (inflation parameter = 3) in order to find natural clusters based on stochastic flow.

## 3. Results

A total of 994 proteins were detected across all analyzed samples; however, 858 86.4%) were further analyzed as they were identified across a sufficient number of samples per group.

**Figure 2:**
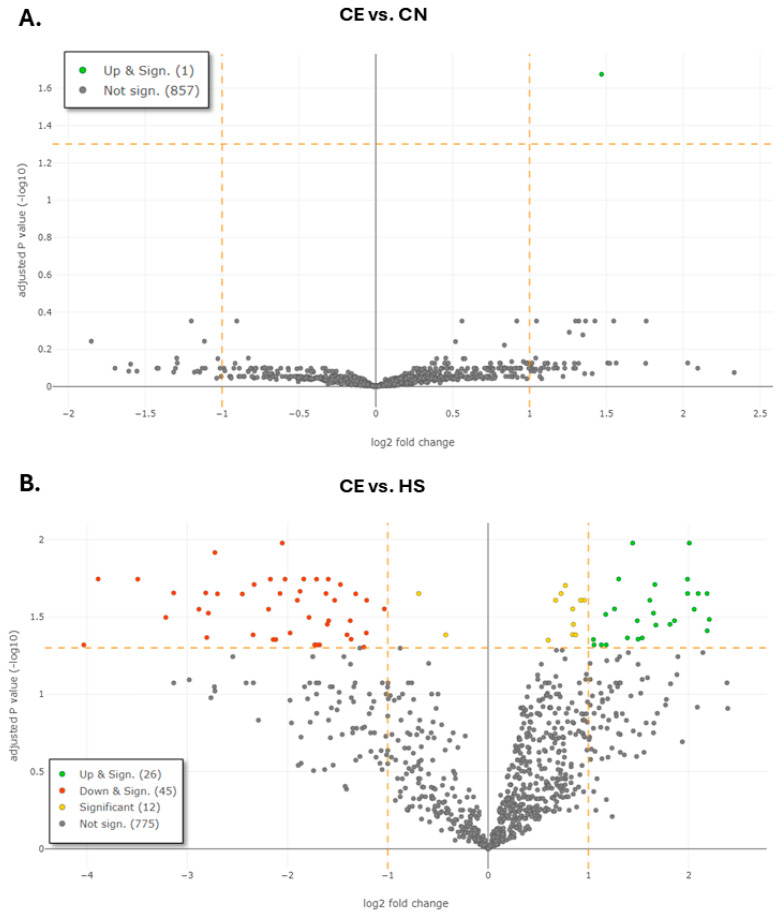
Volcano plots showing differences in protein expression levels across the compared subject groups: (**A**) Comparison of patients with COPD and emphysema (CE) and patients with COPD, without emphysema (CN). (**B**) Comparison of patients with COPD and emphysema (CE) and healthy smokers (HS). Significantly upregulated differentially expressed proteins (DEPs) are depicted in green; significantly downregulated DEPs are depicted in red. Proteins that achieved significance threshold, but not the fold-change threshold, are depicted in yellow. Proteins which are not significant are depicted in grey.

There was a single statistically significantly upregulated DEP when comparing COPD with emphysema and COPD without emphysema (CE vs. CN): NADP-dependent malic enzyme (NADP-ME) (FC = 2.78, *p* = 0.02). When using this approach, we did not identify statistically significant downregulated DEPs (Figure 2A).

When comparing the CE group with healthy smokers (CE vs. HS), we identified 26 upregulated and 45 downregulated DEPs (Table 2 and Table S1, Figure 2B). The top three overabundant proteins (with the highest fold change) were fibroblast growth factor receptor 1 (FGFR-1) (FC 4.61, *p* = 0.03), V-type proton ATPase subunit S1 (FC 4.55, *p* = 0.039) and mannosyl-oligosaccharide glucosidase (FC 4.54, *p* = 0.022).

**Figure 3:**
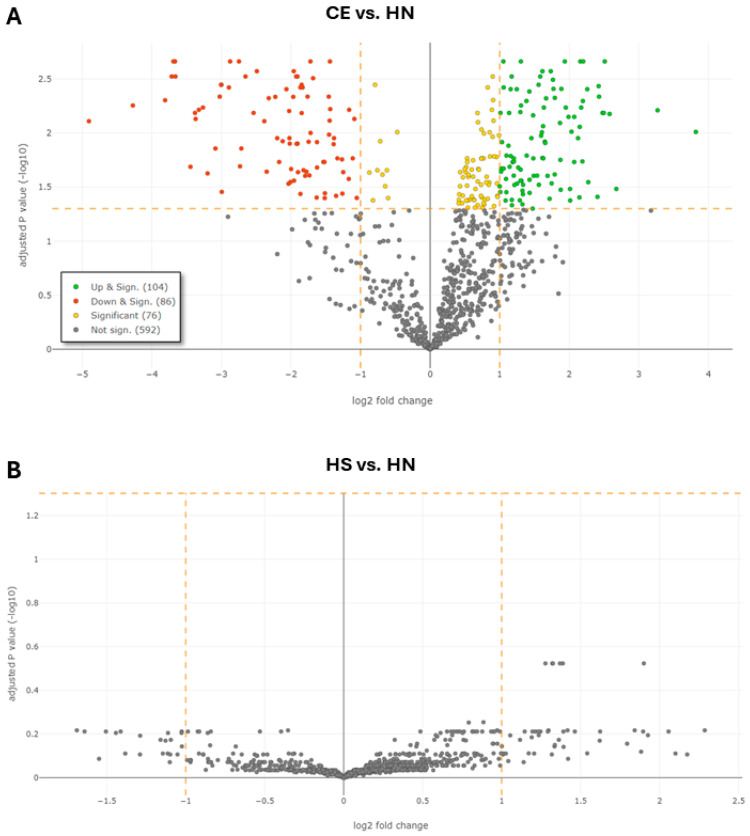
Volcano plots showing differences in protein expression levels across the compared subject groups: (**A**) Comparison of patients with COPD and emphysema (CE) and healthy never-smokers (HN). (**B**) Comparison of healthy smokers (HS) and healthy never-smokers (HN). Significantly upregulated differentially expressed proteins (DEPs) are depicted in green; significantly downregulated DEPs are depicted in red. Proteins that achieved the significance threshold, but not the fold-change threshold, are depicted in yellow. Proteins which are not significant are depicted in grey.

For the comparison of CE with healthy never-smokers (CE vs. HN), we identified 104 upregulated and 86 downregulated proteins (Table 3 and Table S2, Figure 3A). The most overabundant DEPs were plastin-1 (FC 14.11, *p* = 0.009), pulmonary surfactant-associated protein A1 (SP-A1) (FC 9.64, *p* = 0.006) and apolipoprotein(a) (FC 6.39, *p* = 0.03). Importantly, several immunoglobulin components were detected as significantly downregulated DEPs in both CE vs. HS and CE vs. HN contrasts.

**Figure 4:**
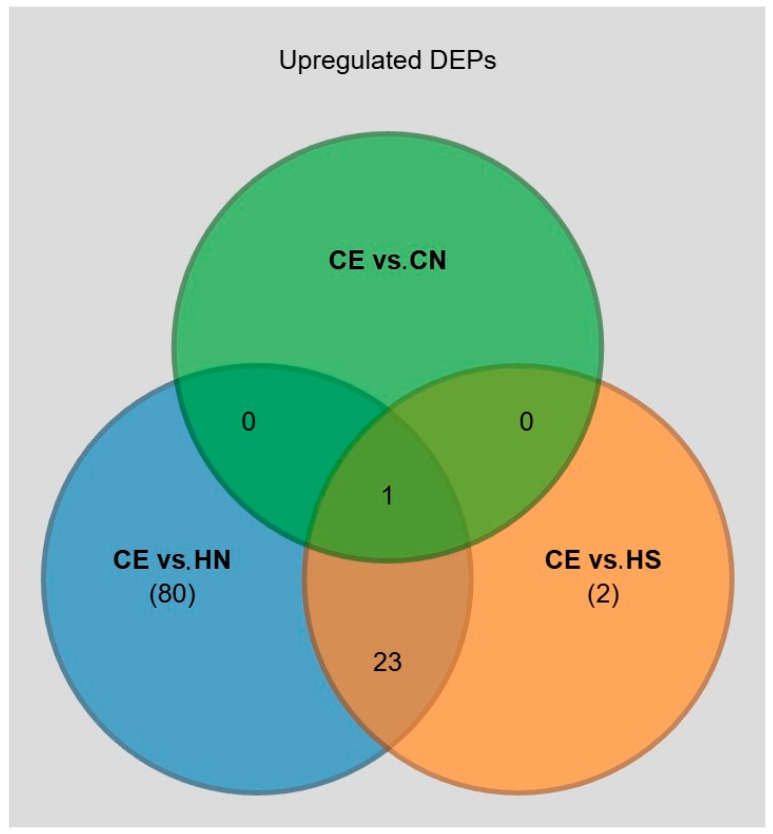
Venn diagram of upregulated proteins across the compared subject groups. The central protein shared across all analyzed groups is NADPH-dependent malic enzyme. CE—patients with emphysema and CODP. CN—patients with COPD without emphysema. HS—healthy smokers. HN—healthy never-smokers.

Using this approach, we did not identify statistically significant DEPs between the two subgroups of healthy individuals (Figure 3B). The majority of upregulated DEPs in the CE vs. HS contrast were also identified in the CE vs. HN contrast (Figure 4). DEPs derived from the comparison between CN and HN is depicted in Supplementary Table S3.

Interestingly, in addition to being a statistically significantly upregulated DEP in the CE vs. CN contrast, NADP-ME was also identified as a significantly upregulated DEP in CE vs. HS (FC = 2.466, *p* = 0.018) and in CE vs. HN (FC = 2.198, *p* = 0.065) (Figure 4).

Overexpression analysis (ORA) for all DEPs (as well as for only upregulated and only downregulated) for CE vs. HN is depicted in Supplementary Table S4. Notably, serine-type endopeptidase activity, insulin-like growth factor I binding, fibronectin binding and transmembrane receptor protein tyrosine kinase activity were among the identified functions. Upon analysis of only upregulated DEPs, thioredoxin peroxidase activity, fibronectin binding, insulin-like growth factor I and II binding and signaling receptor binding were identified to be significantly enriched functions (full list in Supplementary Table S5). Interestingly, processes such as adaptive immune response, classical pathway of complement activation and intermediate filament organization were identified when only downregulated DEPs were analyzed (Supplementary Table S6).

**Figure 5:**
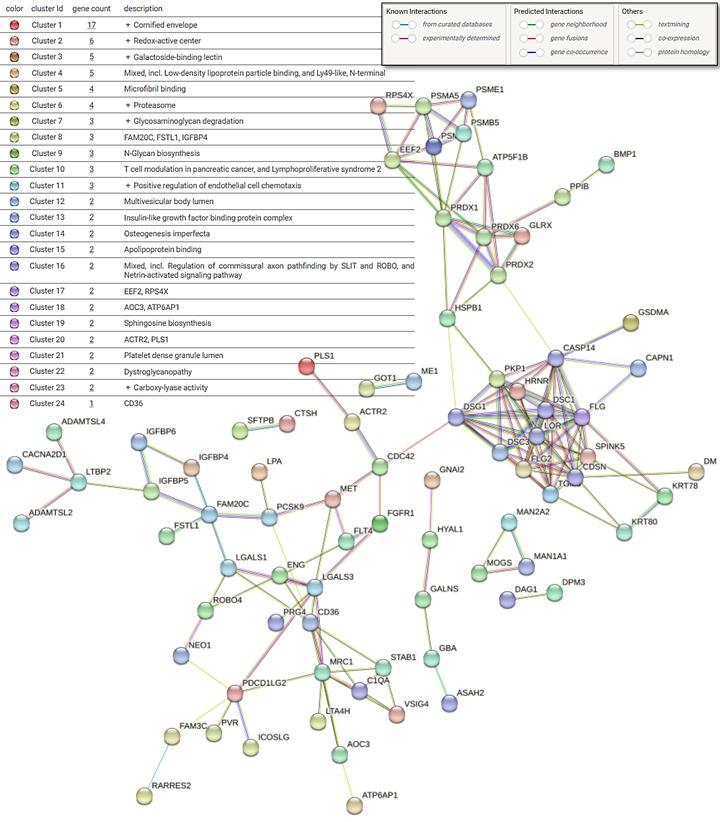
A protein–protein interaction network based on the upregulated differentially expressed proteins derived from the comparison of patients with COPD and emphysema (CE) and healthy never-smokers (HN). Cluster edges are represented with dotted lines. Disconnected nodes are hidden from the network. Created using STRING 12.0.

Additionally, STRING analysis of a protein–protein interaction network from the upregulated DEPs for CE vs. HN revealed several functional clusters of interconnected proteins (Figure 5). MCL cluster analysis revealed 24 distinct clusters, including redox-active center, galactoside-binding lectin, proteasome, insulin-like growth factor binding protein complex, microfibril binding and glycosaminoglycan degradation. Protein–protein interaction of downregulated DEPs is depicted in Supplementary Figure S1. MCL cluster analysis of downregulated DEPs revealed six distinct clusters, including immunoglobulin complex, scavenging by B class receptors, complement pathway and molecules associated with elastic fibers.

## 4. Discussion

We conducted a small shotgun LC-MS-based proteomics study in order to find potential plasma biomarkers of emphysema by comparing patients with stable symptomatic COPD (Gold 2B (2023) [16]) that have emphysema detected on chest CT to those that do not, as well as by comparing them with healthy active smokers and healthy never-smokers.

Our study found (cytosolic) NADP-ME (encoded by the *ME1* gene) to be upregulated in emphysema patients (CE) compared to all other study groups (CN, HS and HN). Namely, NADP-ME catalyzes reversible oxidative decarboxylation of malate to pyruvate, resulting in the reduction of NADP^+^ to NADPH, meaning it is a crucial enzyme of intermediary metabolism and redox homeostasis [30,31].

Cigarette smoke increases oxidative stress levels in COPD patients. Specifically, reactive oxygen and nitrogen species (ROS and RNS) activate the NF-κB pathway with a consequent increase in pro-inflammatory cytokines and recruitment of inflammatory cells, which further generate ROS and increase the oxidative stress burden [32]. NAPDH is a substrate for inducible nitric oxide synthase (iNOS) involved in the synthesis of NO and a substrate for NADPH oxidase (NOX), which leads to the synthesis of ROS through the generation of superoxide anions [31,33]. Increased ROS levels in the lungs increase inflammation, accelerate aging and cellular senescence, increase the potential of development of autoantibodies by protein carbonylation, and cause DNA damage [33]. Additionally, increased iNOS levels have been implicated in the pathogenesis of cigarette-smoke-induced emphysema, and it has been observed that mice with iNOS knockout were protected against emphysema development [33,34].

Another important biological role of NADP-ME (ME1) is its association with resistance to anoikis, a caspase-dependent programmed cell death, which is induced by the loss of cell contacts to the extracellular matrix or to other cells [35,36,37,38]. Anoikis is considered to be a physiological process which acts to safeguard the organism and to maintain tissue integrity [36,39]. Anoikis resistance has been recognized as an important step in cancer progression and metastasis [36,39,40]. Ryan et al. found a complete downregulation of ME1 RNA transcript in COPD-derived alveolar macrophages [30]. It is possible that plasma NADP-ME may therefore reflect tissue injury and systemic metabolic adaptation. However, Hu et al. recently found ME1 to be overexpressed in lung tissues of COPD mice and peripheral blood mononuclears of COPD patients. The authors also developed a diagnostic model based on transcriptional levels of ME1 and other anoikis resistance-related genes (SLC2A1 and BMP4) that were negatively correlated with emphysema index but positively correlated with airway wall thickness [41]. Additionally, elevated levels of cytosolic NADP-ME in plasma stress the importance of exploring the role of anoikis in emphysema development, as imbalances favoring protease activity that promote excessive ECM degradation create conditions that facilitate anoikis [7,8,42].

Elevated NADP-ME levels have thus far been associated with inflammatory (autoimmune) diseases such as rheumatoid arthritis and systemic lupus erythematosus [31]. Systemic immune dysregulation is also suggested by functional clusters of DEPs in CE vs. HN. We also identified upregulated DEPs such as macrophage mannose receptor 1 and Stabilin-1, which are associated with macrophage scavenging and with Galectin-3, which is associated with macrophage activation [43,44,45]. This may indicate persistent innate immune activation and impaired resolution, consistent with chronic tissue injury and remodeling [46].

Therefore, the biological roles of NADP-ME may span several interconnected levels relevant to emphysema pathogenesis: intermediary metabolism, redox homeostasis, amplifcation of inflammatory signaling cascades and anoikis [7,31,36,38,39,41]. We hypothesize that the disturbance in these pathogenic axes in the setting of emphysema might lead to a release of NADP-ME to systemic circulation and propose that plasma NADP-ME might be a potential plasma biomarker candidate of emphysema. However, further studies with other methods are required to validate our results.

When comparing emphysema patients with healthy individuals, other than NADP-ME, our study revealed several DEPs implicated in the homeostasis of redox processes, such as peroxiredoxin-1, -2, and -6 (CE vs. HN), as well as glutaredoxin-1 (CE vs. HN, CE vs. HS), which is also highlighted by the cluster analysis. Glutaredoxin-1 levels have been found to be increased in induced sputum from COPD patients during acute exacerbations; conversely, lower expression levels were found in the alveolar macrophages of COPD patients than in healthy smokers, and decreased enzymatic activity was demonstrated in mice models of pulmonary fibrosis [47,48,49]. Peroxiredoxin-1 was previously suggested as a potential COPD biomarker, and serum levels of peroxiredoxin-6 were higher in COPD patients and correlated with disease severity [50,51].

Our study also identified BMP1 as a significantly upregulated DEP in CE vs. HN (but not in CN vs. HN; Supplementary Table S1). BMP1 is a zinc metalloproteinase responsible for fibrillar procollagen maturation and processing [52,53]. It is important to remark that our findings reflect circulating plasma abundance, suggesting systemic extracellular matrix remodeling rather than local pulmonary expression. Additionally, BMP1 is responsible for the cleavage of several substrates, including Chordin (BMP-2 and BMP-4 antagonist), myostatin, insulin-like growth factor-binding protein 3 (which can bind and sequester IGF-I and IGF-II) [54]. Interestingly, we identified three insulin-like growth factor-binding proteins (IGFBP4, -5 and -6) to be significantly upregulated DEPs in CE vs. HN (IGF-I and II binding were also identified as significantly enriched processes by ORA). Anastasi et al. found that increasing BMP-1 led to the loss of cell adhesion that depended on thrombospondin-1 (TSP-1). BMP1 cleaved TSP-1, which led to TSP-1-mediated activation of latent transforming growth factor-β (TGF-β), leading to increased signaling through the canonical SMAD pathway [54]. Increased immunohistochemistry levels of BMP1 expression were detected in the fibroblastic foci of IPF patients compared to a minimally present signal in healthy lung samples, and its role was observed in liver and kidney fibrosis, as well [52,55,56,57,58]. Stefano et al. did not find a significant difference in immunohistochemical expression of BMP1 (or BMP2, -7, -9, -10 and noggin) in the bronchial epithelium of COPD patients compared to healthy smokers and non-smokers [59]. However, they found a lower number of BMP4+-stained cells in moderate and severe COPD patients compared to healthy individuals [59]. They also found a significantly increased number of BMPER+ cells in COPD and healthy smokers compared to healthy non-smokers [59]. BMP1 has not, thus far, been heavily implicated in the pathogenesis of emphysema or COPD. However, our identification of BMP1 as a significantly overabundant protein in blood plasma of emphysema patients might open a new research avenue. In addition to finding BMP1, the identification of upregulated ECM proteins like ADAMTSL2, ADAMTSL4 and Tsukushi, accompanied by downregulated fibulin-1, -3 and cartilage intermediate layer protein 1 (CILP-1) in blood plasma, indicate the likely systemic effects of significant ECM perturbation in patients with pulmonary emphysema, accompanied by the above-mentioned possible growth factor sequestration.

Our study has several important limitations: namely, this was a small cross-sectional study with a small sample size. However, participants for this study were controlled by matching the sex, age and comorbidities among all four study groups. Additionally, COPD patient groups (CE and CN groups) were clinically similar: they included Gold 2B patients with stable but symptomatic disease undergoing LAMA + LABA therapy with comparable lung function levels. Smoking levels in terms of pack-years measurement were also comparable between smoking groups (CE, CN, and HS). Due to the fact that this study used non-probabilistic sampling, sampling bias is inherently present. An additional study limitation is the fact that emphysema was not quantified but only qualitatively assessed by a radiologist.

To conclude, our small shotgun proteomic study identified NADPH-ME as a potential emphysema biomarker candidate and put emphasis on the importance of ROS homeostasis and the potential role of anoikis in emphysema pathogenesis. Additionally, we found BMP1 and dysregulated ECM components in plasma, which opens a new research horizon to be explored. Taken together, our findings support the concept of emphysema as a systemic disorder reflected by intertwined metabolic, redox and extracellular matrix perturbations. Finally, due to the fact that our findings are based only on LC-MS data, they require further validation with complementary methods and larger cohorts.

## Figures and Tables

**Table 1:** Participants’ characteristics.

	COPD and Emphysema (CE)	COPD Without Emphysema (CN)	Healthy Smokers (HS)	Healthy Never-Smokers (HN)	Statistic	*p*-Value
N	10	10	10	10	NA	
Female sex	5 (50%)	5 (50%)	5 (50%)	5 (50%)	NA	
Age (mean ± SD)	63 ± 3.8	64.5 ± 3.61	61.5 ± 4.53	63 ± 4.94	F = 0.52	0.673
BMI	25.0 ± 3.36	26.7 ± 1.79	26.8 ± 3.61	25.8 ± 3.12	F = 0.69	0.572
Smoking status	Active	Active	Active	Never	NA	
Smoking years (median Q1–Q3))	45.5 (42.3–47.5)	46.5 (43.3–48.8)	41.5 (40–46.5)	/	χ^2^ = 1.492	0.474
Average packs/day (median Q1–Q3))	1 (1–1.75)	1 (1–1.75)	1 (1–2)	/	χ^2^ = 0.102	0.950
TI (pack/years) (median Q1–Q3))	48 (43.5–72.5)	50.5 (43.3–82.3)	47.5 (40.3–83)	/	χ^2^ = 0.127	0.939
CCI (median (Q1–Q3))	3.5 (3–4)	4 (3.25–4.75)	2.5 (2–3)	2.5 (2–3)	**χ^2^ = 16.9**	**<0.001**
Modified CCI (median (Q1–Q3))	2.5 (2–3)	3 (2.25–3.75)	2.5 (2–3)	2.5 (2–3)	χ^2^ = 2.3	0.51
FEV1 (%) mean ± SD	64.2 ± 7.8	62.2 ± 7.51	93.1 ± 10.2	95 ± 11.6	**F = 36.4**	**<0.001**
FVC (%) mean ± SD	81.7 ± 11.4	82.3 ± 11.2	96.7 ± 11.7	97.9 ± 10.8	**F = 5.84**	**0.005**
FEV1/FVC ratio (median (Q1–Q3))	0.61 (0.58–0.65)	0.58 (0.54–0.63)	0.73 (0.72–0.78)	0.77 (0.73–0.79)	**χ^2^ = 30**	**<0.001**
CAT score mean ± SD	22.4 ± 5.97	25 ± 3.87	NA		F = 15.4	0.14

BMI—body mass index; CCI—Charlson’s comorbidity index; modified CCI—Charlson’s comorbidity index when “chronic pulmonary disease” was left out from the score; FEV1—forced expiratory volume in 1 s; FVC—forced vital capacity; SD—standard deviation; Q1—first quartile; Q3—third quartile; TI—tobacco index. Statistically significant results are highlighted in bold.

**Table 2:** List of statistically significant upregulated proteins in patients with COPD and emphysema (CE) compared to healthy smokers (HS).

Accession (UNIPROT ID)	Name	Fold Change	Adjusted *p*-Value
P11362	Fibroblast growth factor receptor 1 (FGFR-1)	4.613141	0.032777
Q15904	V-type proton ATPase subunit S1	4.546022	0.038782
Q13724	Mannosyl-oligosaccharide glucosidase	4.541745	0.022314
Q08188	Protein-glutamine gamma-glutamyltransferase E	4.271874	0.022314
O75493	Carbonic anhydrase-related protein 11 (carbonic anhydrase-related protein 2)	4.155870	0.028208
P60985	Keratinocyte differentiation-associated protein	4.017137	0.010512
P42357	Histidine ammonia-lyase (Histidase)	3.970438	0.022314
P13639	Elongation factor 2 (EF-2)	3.966499	0.018004
P35754	Glutaredoxin-1 (Thioltransferase-1)	3.625121	0.033438
Q86YZ3	Hornerin	3.515543	0.035270
Q5D862	Filaggrin-2	3.188284	0.035684
P08581	Hepatocyte growth factor receptor (HGF receptor)	3.166260	0.019505
Q13835	Plakophilin-1 (band 6 protein) (B6P)	3.136209	0.029858
P28066	Proteasome subunit alpha type-5	3.057153	0.024636
O75340	Programmed cell death protein 6	2.900189	0.043178
P30043	Flavin reductase (NADPH)	2.819734	0.044065
Q6E0U4	Dermokine (epidermis-specific secreted protein SK30/SK89)	2.804329	0.033438
O95497	Pantetheinase	2.716129	0.010512
Q92520	Protein FAM3C	2.618784	0.043178
P48163	NADP-dependent malic enzyme	2.465638	0.017974
A1L4H1	Soluble scavenger receptor cysteine-rich domain-containing protein SSC5D	2.395466	0.028038
P34096	Ribonuclease 4 (RNase 4)	2.262213	0.047869
Q6UX71	Plexin domain-containing protein 2	2.254091	0.030421
P16671	Platelet glycoprotein 4	2.186376	0.047869
Q16853	Amine oxidase [copper-containing] 3	2.080022	0.047869
P28074	Proteasome subunit beta type-5	2.071303	0.044227

**Table 3:** List of statistically significant upregulated proteins in patients with COPD and emphysema (CE) compared to healthy non-smokers (HN).

Accession (UNIPROT ID)	Name	Fold Change	Adjusted *p*-Value
Q14651	Plastin-1 (Intestine-specific plastin)	14.117780	0.009773
Q8IWL2;Q8IWL1	Pulmonary surfactant-associated protein A1	9.640833	0.006159
P08519	Apolipoprotein(a)	6.394886	0.03285
Q96QA5	Gasdermin-A (Gasdermin-1)	5.976723	0.00666
P07988	Pulmonary surfactant-associated protein B (SP-B)	5.696672	0.002175
P11362	Fibroblast growth factor receptor 1 (FGFR-1)	5.643583	0.006516
Q9P2X0	Dolichol-phosphate mannosyltransferase subunit 3	5.574667	0.006516
O75493	Carbonic anhydrase-related protein 11 (Carbonic anhydrase-related protein 2)	5.380649	0.004603
Q08188	Protein-glutamine gamma-glutamyltransferase E	5.359528	0.003685
P49721	Proteasome subunit beta type-2	5.298383	0.038909
P20930	Filaggrin	4.834271	0.02844
P09668	Pro-cathepsin H	4.785878	0.003927
Q9BQ51	Programmed cell death 1 ligand 2 (PD-1 ligand 2) (PD-L2)	4.594920	0.002175
Q86YZ3	Hornerin	4.581005	0.005055
P01718	Immunoglobulin lambda variable 3-27 (Ig lambda chain V-IV region Kern)	4.560397	0.0183
P35754	Glutaredoxin-1 (Thioltransferase-1)	4.504360	0.005804
P08581	Hepatocyte growth factor receptor (HGF receptor)	4.467583	0.002175
Q9Y279	V-set and immunoglobulin domain-containing protein 4 (protein Z39Ig)	4.439814	0.008761
P62701	Small ribosomal subunit protein eS4, X isoform	4.423870	0.039415
Q9NQ38	Serine protease inhibitor Kazal-type 5	4.363249	0.011153
Q9BXR6	Complement factor H-related protein 5 (FHR-5)	4.204383	0.007082
P22692	Insulin-like growth factor-binding protein 4 (IBP-4) (IGF-binding protein 4) (IGFBP-4)	4.201328	0.018519
P04899	Guanine nucleotide-binding protein G(i) subunit alpha-2	4.091585	0.009713
Q14213	Interleukin-27 subunit beta	4.037665	0.042017
P23381	Tryptophan--tRNA ligase, cytoplasmic	4.031435	0.032897
P42357	Histidine ammonia-lyase (Histidase)	3.970438	0.007082
Q9NS71	Gastrokine-1	3.863179	0.005804
P34096	Ribonuclease 4 (RNase 4) (EC 3.1.27.-)	3.826387	0.002175
Q6E0U4	Dermokine	3.698261	0.003898
Q15904	V-type proton ATPase subunit S1	3.673108	0.033427
Q8WUA8	Tsukushi (E2-induced gene 4 protein) (leucine-rich repeat-containing protein 54)	3.662489	0.017682
Q5D862	Filaggrin-2 (FLG-2)	3.649061	0.008073
Q92520	Protein FAM3C	3.465187	0.004726
P61160	Actin-related protein 2 (actin-like protein 2)	3.461103	0.024141
Q99969	Retinoic acid receptor responder protein 2 (chemerin)	3.417870	0.003898
P09960	Leukotriene A-4 hydrolase (LTA-4 hydrolase)	3.400529	0.003212
P30046	D-dopachrome decarboxylase	3.354550	0.029182
O75144	ICOS ligand (B7 homolog 2) (CD antigen CD275)	3.325853	0.002677
P17538;Q6GPI1	Chymotrypsinogen B	3.287377	0.040561
P17174	Aspartate aminotransferase	3.220944	0.024876
P15151	Poliovirus receptor (CD antigen CD155)	3.175410	0.004771
P13639	Elongation factor 2 (EF-2)	3.147544	0.012351
Q13835	Plakophilin-1 (band 6 protein) (B6P)	3.136209	0.011997
O75356	Nucleoside diphosphate phosphatase ENTPD5	3.106214	0.005804
P23284	Peptidyl-prolyl cis-trans isomerase B	3.087764	0.017113
P28066	Proteasome subunit alpha type-5	3.081938	0.00863
P32119	Peroxiredoxin-2	3.063080	0.016982
P24593	Insulin-like growth factor-binding protein 5 (IGFBP-5)	3.062092	0.002677
Q06830	Peroxiredoxin-1	3.044709	0.009841
P30041	Peroxiredoxin-6	3.022820	0.010595
Q9NZK5	Adenosine deaminase 2	2.996210	0.002997
P11277	Spectrin beta chain, erythrocytic	2.955315	0.030042
A1L4H1	Soluble scavenger receptor cysteine-rich domain-containing protein SSC5D	2.946750	0.003579
P60953	Cell division control protein 42 homolog	2.902005	0.024797
Q12794	Hyaluronidase-1	2.805678	0.024876
P17813	Endoglin (CD antigen CD105)	2.795934	0.017113
P34059	N-acetylgalactosamine-6-sulfatase (chondroitinsulfatase)	2.790865	0.00686
Q13724	Mannosyl-oligosaccharide glucosidase	2.776572	0.049997
Q12841	Follistatin-related protein 1 (follistatin-like protein 1)	2.742668	0.011153
Q8WZ75	Roundabout homolog 4 (magic roundabout)	2.695723	0.03573
Q8N1N4	Keratin, type II cytoskeletal 78	2.693386	0.02702
Q6UX71	Plexin domain-containing protein 2	2.687549	0.004123
P06576	ATP synthase F(1) complex subunit beta, mitochondrial	2.617451	0.016173
Q16853	Amine oxidase (vascular adhesion protein 1) (VAP-1)	2.611125	0.005055
P04792	Heat shock protein beta-1 (HspB1)	2.525208	0.030042
Q6UY14	ADAMTS-like protein 4 (ADAMTSL-4)	2.522055	0.036864
P13497	Bone morphogenetic protein 1 (BMP-1)	2.509089	0.034063
P35916	Vascular endothelial growth factor receptor 3 (VEGFR-3)	2.508936	0.045614
P18850	Cyclic AMP-dependent transcription factor ATF-6 alpha (activating transcription factor 6 alpha)	2.484812	0.027612
Q9NY15	Stabilin-1	2.473338	0.005556
P22897	Macrophage mannose receptor 1 (CD antigen CD206)	2.469403	0.002175
P04062	Lysosomal acid glucosylceramidase	2.464167	0.021779
Q6KB66	Keratin, type II cytoskeletal 80	2.461539	0.027561
P28074	Proteasome subunit beta type-5	2.457306	0.006389
O75340	Programmed cell death protein 6	2.445039	0.041905
Q92484	Cyclic GMP-AMP phosphodiesterase SMPDL3A	2.405827	0.02085
Q14767	Latent-transforming growth factor beta-binding protein 2 (LTBP-2)	2.380375	0.029284
P49641	Alpha-mannosidase 2x	2.370262	0.003927
O75828;P16152	Carbonyl reductase [NADPH] 3	2.317665	0.030166
Q8IWV2	Contactin-4 (brain-derived immunoglobulin superfamily protein 2) (BIG-2)	2.304669	0.0183
Q86TH1	ADAMTS-like protein 2 (ADAMTSL-2)	2.302448	0.017113
Q8TDL5	BPI fold-containing family B member 1	2.300571	0.024797
O15335	Chondroadherin (cartilage leucine-rich protein)	2.275375	0.018601
P09382	Galectin-1 (Gal-1)	2.259794	0.046466
P17931	Galectin-3 (Gal-3)	2.257579	0.041507
Q8NBP7	Proprotein convertase subtilisin/kexin type 9	2.253341	0.002997
P00918	Carbonic anhydrase 2 (CA-II)	2.233674	0.016313
P54289	Voltage-dependent calcium channel subunit alpha-2/delta-1	2.230589	0.003927
Q8IXL6	Extracellular serine/threonine protein kinase FAM20C	2.208789	0.020499
P48163	NADP-dependent malic enzyme (NADP-ME) (EC 1.1.1.40) (malic enzyme 1)	2.197616	0.006516
Q86U17	Serpin A11	2.183316	0.013121
Q14574	Desmocollin-3 (Desmocollin-4) (HT-CP)	2.139147	0.016173
P24592	Insulin-like growth factor-binding protein 6 (IBP-6) (IGF-binding protein 6) (IGFBP-6)	2.137108	0.035248
P16671	Platelet glycoprotein 4	2.130166	0.02657
P07384	Calpain-1 catalytic subunit	2.121511	0.042113
Q92859	Neogenin	2.080039	0.003783
Q06323	Proteasome activator complex subunit 1	2.079338	0.021289
P33908	Mannosyl-oligosaccharide 1,2-alpha-mannosidase IA	2.074546	0.002175
O95497	Pantetheinase	2.046274	0.012125
Q9P232	Contactin-3	2.029209	0.021289
Q92954	Proteoglycan 4 (lubricin)	2.027655	0.022776
Q9NR71	Neutral ceramidase	2.015363	0.006516
Q14118	Dystroglycan 1 (dystroglycan)	2.011917	0.035147
P02745	Complement C1q subcomponent subunit A	2.000559	0.031144

## Data Availability

The original data presented in the study are openly available in ProteomeXchange Consortium via the PRIDE partner repository with identifier PXD074107.

## Supplementary Materials

The following supporting information can be downloaded at: https://www.mdpi.com/article/10.3390/diagnostics16060931/s1. Figure S1. A protein-protein interaction network based on the downregulated differentially expressed proteins derived from the comparison of patients with COPD and emphysema (CE) and healthy never-smokers (HN); Table S1. List of statistically significant downregulated proteins in patients with COPD and emphysema (CE), compared to healthy smokers (HS); Table S2. List of statistically significant downregulated proteins in patients with COPD and emphysema (CE), compared to healthy non-smokers (HN); Table S3. List of statistically significant upregulated and downregulated differentially expressed proteins in patients with COPD without emphysema (CN), compared to healthy never-smokers (HN). Table S4. Identified enriched functions and processes through overrepresentation analysis for all (up- and downregulated) differentially expressed proteins in comparison of patients with COPD and emphysema to healthy never-smokers. Table S5. Identified enriched functions and processes through overrepresentation analysis for upregulated differentially expressed proteins in comparison of patients with COPD and emphysema to healthy never-smokers. Table S6. Identified enriched functions and processes through overrepresentation analysis for downregulated differentially expressed proteins in comparison of patients with COPD and emphysema to healthy never-smokers.
