# Supplementary material for: Proteomics-Based Study of Potential Emphysema Biomarkers Reveals Systemic Redox System and Extracellular Matrix Component Dysregulation
Source: Diagnostics (Basel). 2026 Mar 21;16(6):931. doi: 10.3390/diagnostics16060931 (PMC13025604; doi:10.3390/diagnostics16060931)
Supplement: Supplementary file 1 [file diagnostics-16-00931-s001.zip › diagnostics-4180500-supplementary.pdf]

Proteomics-based study of potential emphysema biomarkers reveals systemic redox-system and extracellular matrix component dysregulation

Grgur Salai, Ruđer Novak, Stela Hrkač, Václav Pustka, David Potěšil, Zbyněk Zdráhal, Đivo Ljubičić, Lovorka Grgurević

Supplementary material:

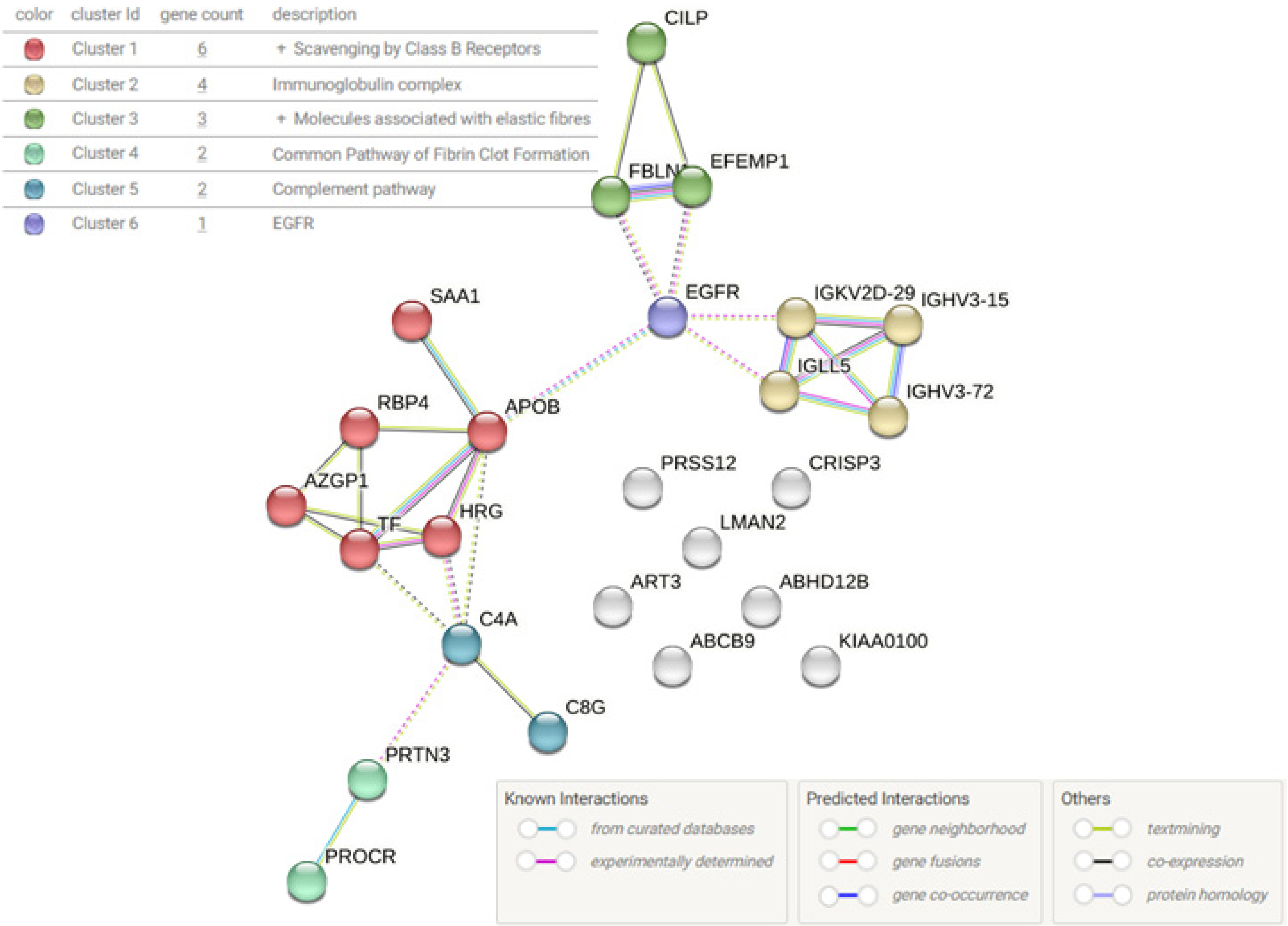

Supplementary Figure S1. A protein-protein interaction network based on the downregulated differentially expressed proteins derived from the comparison of patients with COPD and emphysema (CE) and healthy never-smokers (HN). Cluster edges are represented with dotted lines. Created using STRING 12.0.

Supplementary Table S1. List of statistically significant downregulated proteins in patients with COPD and emphysema (CE), compared to healthy smokers (HS).

Accession number (UNIPROT ID)	Name	Fold change	Adjusted p-value
P01700	Immunoglobulin lambda variable 1-47	0.241134	0.010512
P06310	Immunoglobulin kappa variable 2-30	0.151371	0.012099
Q14667	Bridge-like lipid transfer protein family member 2	0.067548	0.017974
P01717	Immunoglobulin lambda variable 3-25	0.279714	0.017974
P01780	Immunoglobulin heavy variable 3-7	0.222133	0.018004
A0A0A0MRZ8; P04433	Immunoglobulin kappa variable 3D-11	0.305562	0.018004
P01593;P01594	Immunoglobulin kappa variable 1D-33	0.331367	0.018004
A0A075B6H7	Probable non-functional immunoglobulin kappa variable 3-7	0.088823	0.018004
A0A0J9YY99	Ig-like domain-containing protein	0.245799	0.018004
P31946	14-3-3 protein beta/alpha	0.360454	0.019505
A0A0C4DH73; P01611	Immunoglobulin kappa variable 1-12	0.198519	0.019505
P01715	Immunoglobulin lambda variable 3-1	0.272754	0.021578
A0A0C4DH34	Immunoglobulin heavy variable 4-28	0.11385	0.022105
A0A075B6I9	Immunoglobulin lambda variable 7-46	0.142108	0.022105
P01857	Immunoglobulin heavy constant gamma 1 (Ig gamma-1 chain C region)	0.326251	0.022314
A0A075B6S2	Immunoglobulin kappa variable 2D-29	0.23745	0.022314
P01619	Immunoglobulin kappa variable 3-20	0.400592	0.022383
A0A087WSX0	Immunoglobulin lambda variable 5-45	0.154077	0.022383
A0A0B4J1X5	Immunoglobulin heavy variable 3-74	0.182985	0.022438
A0A075B6S5	Immunoglobulin kappa variable 1-27	0.432043	0.024636
A0A075B6R9	Probable non-functional immunoglobulin kappa variable 2D-24	0.267653	0.024636
P06312	Immunoglobulin kappa variable 4-1	0.346235	0.024636
P56730	Neurotrypsin	0.488375	0.028059
A0A0B4J1V2	Immunoglobulin heavy variable 2-26	0.219322	0.028157
P0DOY3	Immunoglobulin lambda constant 3	0.135562	0.028157
O14950; P19105	Myosin regulatory light chain 12B	0.144711	0.029858
P25311	Zinc-alpha-2-glycoprotein	0.107834	0.031835
P02743	Serum amyloid P-component (SAP)	0.290106	0.031835
O75339	Cartilage intermediate layer protein 1 (CILP-1)	0.332254	0.033438

A0A0A0MS15	Immunoglobulin heavy variable 3-49	0.38574	0.033438
P01602	Immunoglobulin kappa variable 1-5	0.329247	0.03527
A0A0B4J1U7	Immunoglobulin heavy variable 6-1	0.2545	0.04007
P01833	Polymeric immunoglobulin receptor (PIgR) (Poly-Ig receptor)	0.431052	0.04007
P01701	Immunoglobulin lambda variable 1-51	0.377209	0.041276
P02753	Retinol-binding protein 4	0.197152	0.041276
A0A075B6W8	T cell receptor alpha joining 17	0.143176	0.042907
Q9UNN8	Endothelial protein C receptor	0.388016	0.044065
A0A0C4DH25	Immunoglobulin kappa variable 3D-20	0.226414	0.044227
Q9NP78	ABC-type oligopeptide transporter ABCB9	0.230573	0.044227
P01601	Immunoglobulin kappa variable 1D-16	0.302884	0.047869
P01817	Immunoglobulin heavy variable 2-5	0.312211	0.047869
Q7Z5M8	Protein ABHD12B	0.061186	0.047869
Q12805	EGF-containing fibulin-like extracellular matrix protein 1 (Fibulin-3) (FIBL-3)	0.308234	0.047869
A0A0C4DH31	Immunoglobulin heavy variable 1-18	0.30173	0.047869
A0A087WSY6	Immunoglobulin kappa variable 3D-15	0.423673	0.049492

Supplementary Table S2. List of statistically significant downregulated proteins in patients with COPD and emphysema (CE), compared to healthy non-smokers (HN).

Accession number (UNIPROT ID)	Name	Fold change	Adjusted p-value
P06310	Immunoglobulin kappa variable 2-30	0.136441	0.002175
A0A075B6I9	Immunoglobulin lambda variable 7-46	0.078714	0.002175
A0A075B6S5	Immunoglobulin kappa variable 1-27	0.303586	0.002175
P56730	Neurotrypsin (Serine protease 12)	0.36862	0.002175
P01601	Immunoglobulin kappa variable 1D-16	0.148926	0.002175
A0A087WSX0	Immunoglobulin lambda variable 5-45	0.077508	0.002175
P01780	Immunoglobulin heavy variable 3-	0.17798	0.002677
A0A0A0MRZ8; P04433	Immunoglobulin kappa variable 3D-11	0.257119	0.002677
P01700	Immunoglobulin lambda variable 1-47	0.263328	0.002997
P06312	Immunoglobulin kappa variable 4-1	0.266592	0.002997
P0DOY3	Immunoglobulin lambda constant 3	0.076185	0.002997
A0A0C4DH34	Immunoglobulin heavy variable 4-28	0.079076	0.002997
A0A0C4DH24	Immunoglobulin kappa variable 6-21	0.158975	0.002997
P23142	Fibulin-1 (FIBL-1)	0.311704	0.003109
A0A0C4DH25	Immunoglobulin kappa variable 3D-20	0.124968	0.003579
A0A0A0MTA3	Immunoglobulin kappa joining 5	0.277968	0.003579
A0A075B6P5; P01615	Immunoglobulin kappa variable 2-28; I	0.124645	0.003579
P01857	Immunoglobulin heavy constant gamma 1	0.280827	0.003783
P01859	Immunoglobulin heavy constant gamma 2	0.134728	0.003783
Q9UNN8	Endothelial protein C receptor (CD antigen CD201)	0.274598	0.003783
A0A0J9YY99	Ig-like domain-containing protein	0.23897	0.003927
P02753	Retinol-binding protein 4	0.122969	0.004603
P01717	Immunoglobulin lambda variable 3-25	0.295136	0.004603
P01619	Immunoglobulin kappa variable 3-20	0.364319	0.004609
A0A075B6S2	Immunoglobulin kappa variable 2D-29	0.213958	0.00463
P01715	Immunoglobulin lambda variable 3-1	0.255892	0.00463
A0A075B6S6	Immunoglobulin kappa variable 2D-30	0.200368	0.004752
P25311	Zinc-alpha-2-glycoprotein	0.071388	0.004967
O43790;P78385; P78386;Q14533	Keratin, type II cuticular Hb6	0.051749	0.005556

Q12907	Vesicular integral-membrane protein VIP36 (Glycoprotein GP36b) (VIP36)	0.104072	0.005804
A0A0J9YX35	Immunoglobulin heavy variable 3-64D	0.36965	0.005995
P01834	Immunoglobulin kappa constant (Ig kappa chain C region)	0.446341	0.006095
A0A075B6H7	Probable non-functional immunoglobulin kappa variable 3-7	0.099911	0.006095
A0A075B6R9	Probable non-functional immunoglobulin kappa variable 2D-24	0.245603	0.006259
A0A0B4J1X5	Immunoglobulin heavy variable 3-74	0.172297	0.006516
A0A075B6W8	T cell receptor alpha joining 17	0.096104	0.006516
P01602	Immunoglobulin kappa variable 1-5	0.278095	0.006524
P02787	Serotransferrin (Transferrin) (Beta-1 metal-binding globulin) (Siderophilin)	0.470321	0.007406
Q14667	Bridge-like lipid transfer protein family member 2	0.096724	0.007406
P01593; P01594	Immunoglobulin kappa variable 1D-33 (Ig kappa chain V-I region AG)	0.36749	0.007683
A0A075B7B8	Immunoglobulin heavy variable 3/OR16-12 (non-functional)	0.19173	0.007758
Q7Z5M8	Protein ABHD12B	0.03341	0.007758
P23083	Immunoglobulin heavy variable 1-2 (Ig heavy chain V-I region ND)	0.304	0.00996
A0A087WSY6	Immunoglobulin kappa variable 3D-15	0.365431	0.010335
P01599	Immunoglobulin kappa variable 1-17	0.383931	0.011153
A0A0C4DH67	Immunoglobulin kappa variable 1-8	0.21804	0.011153
A0A075B6S9; P0DSN7	Probable non-functional immunoglobulinn kappa variable 1-37	0.246288	0.011153
B9A064	Immunoglobulin lambda-like polypeptide 5 (G lambda-1)	0.23015	0.01189
A0A0C4DH36	Probable non-functional immunoglobulin heavy variable 3-38	0.298159	0.012047
O75339	Cartilage intermediate layer protein 1 (CILP-1)	0.323976	0.012125
A0A0B4J1V0	Immunoglobulin heavy variable 3-15	0.247087	0.012512
Q12805	EGF-containing fibulin-like extracellular matrix protein 1 (Extracellular protein S1-5) (Fibrillin-like protein) (Fibulin-3) (FIBL-3)	0.266735	0.012512
A0A0C4DH31	Immunoglobulin heavy variable 1-18	0.264428	0.012595

P04430	Immunoglobulin kappa variable 1-16 (Ig kappa chain V-I region BAN)	0.382438	0.01268
O14950; P19105	Myosin regulatory light chain 12B (MLC-2A) (MLC-2)	0.152523	0.013899
Q13508	Ecto-ADP-ribosyltransferase 3	0.117847	0.013899
P01871	Immunoglobulin heavy constant mu (Ig mu chain C region)	0.462754	0.017113
A0A0A0MS15	Immunoglobulin heavy variable 3-49	0.39717	0.017113
P04196	Histidine-rich glycoprotein (Histidine-proline-rich glycoprotein) (HPRG)	0.415759	0.017543
P01861	Immunoglobulin heavy constant gamma 4 (Ig gamma-4 chain C region)	0.345498	0.018519
P06331	Immunoglobulin heavy variable 4-34 (Ig heavy chain V-II region ARH-77)	0.222293	0.018519
P0CG47; P0CG48; P62979; P62987	Polyubiquitin-B [Cleaved into: Ubiquitin]	0.349048	0.018519
P54108	Cysteine-rich secretory protein 3 (CRISP-3)	0.150513	0.020313
P04211	Immunoglobulin lambda variable 7-43 (Ig lambda chain V region 4A)	0.091871	0.020499
P0DJI8	Serum amyloid A-1 protein (SAA)	0.32425	0.020835
A0A087WSZ0	Immunoglobulin kappa variable 1D-8	0.250521	0.02149
A0A0J9YWN2	Immunoglobulin heavy joining 2	0.283231	0.022053
A0A0B4J1U3	Immunoglobulin lambda variable 1-36	0.196274	0.022776
A0A0C4DH73; P01611	Immunoglobulin kappa variable 1-12	0.291803	0.022778
P07360	Complement component C8 gamma chain	0.420977	0.022976
A0A0B4J1V2	Immunoglobulin heavy variable 2-26	0.26816	0.022976
A0A075B6R2	Immunoglobulin heavy variable 4-4	0.10894	0.023633
A0A0A0MT96	Immunoglobulin kappa joining 3	0.300452	0.024644
A0A0A0MT36	Immunoglobulin kappa variable 6D-21	0.287938	0.024876
A0A0B4J1Y9	Immunoglobulin heavy variable 3-72	0.444667	0.02657
A0M8Q6	Immunoglobulin lambda constant 7	0.257745	0.027546
A0A087WW87; P01614	Immunoglobulin kappa variable 2-40	0.248787	0.028408
P68431; P84243; Q16695;	Histone H3.1	0.243993	0.029434

Q5TEC6;<br>Q6NXT2;<br>Q71DI3			
A0A075B6I0	Immunoglobulin lambda variable 8-61	0.125468	0.035046
P01817	Immunoglobulin heavy variable 2-5	0.350666	0.036075
P0C0L4	Complement C4-A	0.417348	0.036333
Q9NP78	ABC-type oligopeptide transporter ABCB9	0.274701	0.036544
A0A0A0MT89	Immunoglobulin kappa joining 1	0.391911	0.038112
P04114	Apolipoprotein B-100 (Apo B-100)	0.322957	0.039509
P00533	Epidermal growth factor receptor	0.48172	0.039859
P24158	Myeloblastin (C-ANCA antigen) (PR3)	0.350072	0.040019

Supplementary Table S3. List of statistically significant upregulated and downregulated differentially expressed proteins in patients with COPD without emphysema (CN), compared to healthy never-smokers (HN).

Upregulated DEPs (CN vs. HN)			
Accession number (UNIPROT ID)	Name	Fold change	Adjusted p-value
P07988	Pulmonary surfactant-associated protein B (SP-B)	6.999677	0.001194
P34096	Ribonuclease 4 (RNase 4)	4.472332	0.00126
Q9BQ51	Programmed cell death 1 ligand 2 (PD-1 ligand 2) (PD-L2)	4.505975	0.00126
P61626	Lysozyme C	2.657928	0.003295
Q86TH1	ADAMTS-like protein 2 (ADAMTSL-2)	3.694688	0.003295
Q9NZK5	Adenosine deaminase 2	3.234133	0.003421
P33908	Mannosyl-oligosaccharide 1.2-alpha-mannosidase IA	2.015966	0.004864
P49641	Alpha-mannosidase 2x	2.486382	0.006947
A1L4H1	Soluble scavenger receptor cysteine-rich domain-containing protein SSC5D (SSc5D)	3.023142	0.006947
Q9Y279	V-set and immunoglobulin domain-containing protein 4 (Protein Z39Ig)	5.921251	0.007616
P34059	N-acetylgalactosamine-6-sulfatase (Chondroitinsulfatase)	3.150649	0.009196
Q08830	Fibrinogen-like protein 1 (Hepatocyte-derived fibrinogen-related protein 1) (HFREP-1)	2.914514	0.009507
Q8TER0	Sushi. nidogen and EGF-like domain-containing protein 1 (Insulin-responsive sequence DNA-binding protein 1) (IRE-BP1)	2.349849	0.009507
Q92520	Protein FAM3C (Interleukin-like EMT inducer)	3.512606	0.010131
Q99969	Retinoic acid receptor responder protein 2 (Chemerin) (RAR-responsive protein TIG2) (Tazarotene-induced gene 2 protein)	3.268804	0.010641
P15151	Poliovirus receptor (Nectin-like protein 5) (NECL-5) (CD antigen CD155)	3.158383	0.010692
Q4LDE5	Sushi. von Willebrand factor type A. EGF and pentraxin domain-containing protein 1	2.798917	0.010692
O75493	Carbonic anhydrase-related protein 11 (CA-RP XI) (CA-XI) (CARP XI) (CARP-2)	5.084519	0.01121
P18428	Lipopolysaccharide-binding protein (LBP)	2.065017	0.011265
P01718	Immunoglobulin lambda variable 3-27 (Ig lambda chain V-IV region Kern)	6.267842	0.011819
O75144	ICOS ligand (B7 homolog 2) (B7-H2) (B7-like protein Gl50) (B7-related protein 1) (B7RP-1) (CD antigen CD275)	2.650018	0.011819
Q9P232	Contactin-3 (Plasmacytoma-associated neuronal glycoprotein)	2.401894	0.011819
Q9NS71	Gastrokine-1 (Protein CA11)	3.81871	0.011819
P22692	Insulin-like growth factor-binding protein 4 (IGFBP-4)	5.886767	0.011819
Q8WWA0	Intelectin-1 (ITLN-1)	3.653425	0.011819

Q9Y240	C-type lectin domain family 11 member A (C-type lectin superfamily member 3)	2.750779	0.011819
P01854	Immunoglobulin heavy constant epsilon (Ig epsilon chain C region)	8.036423	0.012635
Q96CX2	BTB/POZ domain-containing protein KCTD12 (Pfetin)	2.072896	0.013402
Q8IWV2	Contactin-4 (BIG-2)	2.607998	0.015962
O75356	Nucleoside diphosphate phosphatase ENTPD5 (CD39 antigen-like 4)	2.872856	0.015962
P55103	Inhibin beta C chain (Activin beta-C chain)	2.10294	0.016262
P09382	Galectin-1 (Gal-1)	3.087183	0.018043
P09960	Leukotriene A-4 hydrolase	2.540062	0.021888
Q9NQ38	Serine protease inhibitor Kazal-type 5 (Lympho-epithelial Kazal-type-related inhibitor) (LEKTI)	4.212885	0.021888
Q8WVN6	Secreted and transmembrane protein 1 (Protein K-12)	3.240303	0.021888
Q6UX71	Plexin domain-containing protein 2	2.215078	0.022711
Q8WZ75	Roundabout homolog 4 (Magic roundabout)	3.255762	0.026249
P24592	Insulin-like growth factor-binding protein 6 (IBP-6) (IGF-binding protein 6) (IGFBP-6)	2.397446	0.032472
Q12841	Follistatin-related protein 1	2.444262	0.037181
P58166	Inhibin beta E chain (Activin beta-E chain)	2.70693	0.037211
Q10471	Polypeptide N-acetylgalactosaminyltransferase	3.983768	0.037211
Q9BXR6	Complement factor H-related protein 5 (FHR-5)	3.222843	0.038614
Q14574	Desmocollin-3 (Cadherin family member 3) (Desmocollin-4) (HT-CP)	2.011886	0.039874
Q8IWL2; Q8IWL1	Pulmonary surfactant-associated protein A1 (PSP-A) Pulmonary surfactant-associated protein A2 (PSP-A)	5.817967	0.040875
P12318	Low affinity immunoglobulin gamma Fc region receptor II-a	3.340755	0.040878
Q16853	Amine oxidase [copper-containing] 3	2.031976	0.04214
Q6UY14	ADAMTS-like protein 4 (ADAMTSL-4)	2.672027	0.046532
P06576	ATP synthase F(1) complex subunit beta. mitochondrial (ATP synthase F1 subunit beta)	2.334007	0.046785
Downregulated DEPs (CN vs. HN)			
Accession number (UNIPROT ID)	Name	Fold change	Adjusted p-value
A0A0C4DH24	Immunoglobulin kappa variable 6-21	0.134609	0.003421
A0A087WSX0	Immunoglobulin lambda variable 5-45	0.090628	0.004189
A0A075B6R9	Probable non-functional immunoglobulin kappa variable 2D-24	0.200522	0.006947
Q7Z5M8	Protein ABHD12B (EC 3.-.-.-) (Abhydrolase domain-containing protein 12B)	0.016912	0.006947
A0A075B7B8	Immunoglobulin heavy variable 3/OR16-12 (non-functional)	0.141774	0.007409
A0A075B6S5	Immunoglobulin kappa variable 1-27	0.364985	0.007683
A0A075B6S6	Immunoglobulin kappa variable 2D-30	0.189361	0.009196
A0A075B6P5; P01615	Immunoglobulin kappa variable 2-28	0.145534	0.010692

O43790; P78385; P78386; Q14533	Keratin. type II cuticular Hb6	0.050979	0.01121
P06310	Immunoglobulin kappa variable 2-30	0.204626	0.011819
A0A075B6I9	Immunoglobulin lambda variable 7-46	0.142797	0.011819
P01700	Immunoglobulin lambda variable 1-47	0.338587	0.015962
A0A0C4DH25	Immunoglobulin kappa variable 3D-20	0.177605	0.017032
P01859	Immunoglobulin heavy constant gamma 2	0.183859	0.017209
A0A0C4DH34	Immunoglobulin heavy variable 4-28	0.131071	0.017209
A0A0A0MRZ8; P04433	Immunoglobulin kappa variable 3D-11	0.359886	0.018043
P23142	Fibulin-1 (FIBL-1)	0.395604	0.018043
A0A0C4DH67	Immunoglobulin kappa variable 1-8	0.210532	0.018043
P01619	Immunoglobulin kappa variable 3-20	0.419162	0.018994
A0A0J9YX35	Immunoglobulin heavy variable 3-64D	0.410973	0.020623
P06312	Immunoglobulin kappa variable 4-1 (Ig kappa chain V-IV region B17) (Ig kappa chain V-IV region JI) (Ig kappa chain V-IV region Len)	0.362662	0.020958
Q9BY67	Cell adhesion molecule 1	0.339744	0.020958
P0DOY3	Immunoglobulin lambda constant 3	0.137858	0.020979
P01780	Immunoglobulin heavy variable 3-7	0.283791	0.021859
P01871	Immunoglobulin heavy constant mu	0.44639	0.021888
P01601	Immunoglobulin kappa variable 1D-16	0.264419	0.021888
A0A0C4DH33	Immunoglobulin heavy variable 1-24	0.274101	0.021888
P02753	Retinol-binding protein 4	0.181726	0.022873
A0A0B4J1V0	Immunoglobulin heavy variable 3-15	0.253969	0.022873
A0A0C4DH31	Immunoglobulin heavy variable 1-18	0.276157	0.02581
A0A0B4J1Y9	Immunoglobulin heavy variable 3-72	0.413861	0.032083
P01599	Immunoglobulin kappa variable 1-17 (Ig kappa chain V-I region Gal) (Ig kappa chain V-I region WEA)	0.418782	0.032472
P04430	Immunoglobulin kappa variable 1-16 (Ig kappa chain V-I region BAN)	0.407641	0.032472
P07360	Complement component C8 gamma chain	0.405185	0.032887
P01593; P01594	Immunoglobulin kappa variable 1D-33 (Ig kappa chain V-I region AG) Immunoglobulin kappa variable 1-33 (Ig kappa chain V-I region AU) (Ig kappa chain V-I region Ka)	0.42726	0.033131
P10599	Thioredoxin (Trx) (ATL-derived factor) (ADF)	0.393702	0.033131
P25311	Zinc-alpha-2-glycoprotein (Zn-alpha-2-GP)	0.137246	0.039874
P01715	Immunoglobulin lambda variable 3-1 (Ig lambda chain V-IV region Bau)	0.368311	0.040878
Q12907	Vesicular integral-membrane protein VIP36 (Glycoprotein GP36b)	0.177644	0.040878
P01602	Immunoglobulin kappa variable 1-5	0.365638	0.041454
Q15828	Cystatin-M (Cystatin-6) (Cystatin-E)	0.406633	0.041775
P23083	Immunoglobulin heavy variable 1-2	0.370215	0.04185

B9A064	Immunoglobulin lambda-like polypeptide 5 (G lambda-1)	0.292545	0.046785
P01597; P04432	Immunoglobulin kappa variable 1-39	0.250954	0.046785
A0A075B6H7	Probable non-functional immunoglobulin kappa variable 3-7	0.183838	0.049646

Supplementary Table S4. Identified enriched functions and processes through overrepresentation analysis for all (up- and downregulated) differentially expressed proteins in comparison of patients with COPD and emphysema to healthy never-smokers.

Function description	FDR	Number of genes
Structural constituent of chromatin	1.75e-14	18
Structural molecule activity	8.18e-14	36
Protein heterodimerization activity	1.04e-08	21
Structural constituent of skin epidermis	3.07e-06	8
Protein dimerization activity	2.24e-05	29
Growth factor binding	0.00013	10
Cadherin binding	0.00013	15
Calcium ion binding	0.00029	21
Protein binding	0.00029	90
Cell adhesion molecule binding	5.00E-04	18
Fibronectin binding	0.0038	5
Protein tag	0.0038	4
Signaling receptor binding	0.0047	29
Antigen binding	0.013	6
Glycosaminoglycan binding	0.0142	10
Thioredoxin peroxidase activity	0.0182	3
Scavenger receptor activity	0.0194	5
Transmembrane receptor protein kinase activity	0.0201	6
Insulin-like growth factor II binding	0.0203	3
Cargo receptor activity	0.0203	6
Complement binding	0.0212	4
Heparin binding	0.024	8
Serine-type endopeptidase activity	0.0252	8

Laminin binding	0.0252	4
Insulin-like growth factor I binding	0.0262	3
Transmembrane receptor protein tyrosine kinase activity	0.0403	5
Process description	FDR	Number of genes
Nucleosome assembly	4.18e-10	16
DNA replication-dependent chromatin assembly	8.02e-09	10
Regulation of gene expression. epigenetic	2.18e-06	12
Telomere organization	2.8e-06	12
Immune system process	1.92e-05	43
Epithelium development	6.91e-05	28
Skin development	7.84e-05	14
Immune response	0.00012	31
Epithelial cell differentiation	0.00022	20
Adaptive immune response	0.00037	15
Tissue development	0.00038	35
Epidermis development	0.00049	14
Keratinization	0.00059	8
Complement activation	0.00088	7
Epidermal cell differentiation	0.00091	11
Biological process involved in interspecies interaction between organisms	0.00095	31
Intermediate filament organization	0.0018	7
Humoral immune response	0.0019	12
Keratinocyte differentiation	0.0019	9
Regulation of body fluid levels	0.0019	14
Response to other organism	0.0022	28

Defense response	0.0048	28
Response to external stimulus	0.0057	39
Regulation of water loss via skin	0.0057	5
Multicellular organismal homeostasis	0.0067	13
Defense response to other organism	0.0092	22
Complement activation. classical pathway	0.0127	5
Digestion	0.0151	7
Regulation of endothelial cell chemotaxis	0.0153	4
Positive regulation of epithelial cell migration	0.0169	8
Response to chemical	0.021	54
Regulation of insulin-like growth factor receptor signaling pathway	0.022	4
Immune effector process	0.0255	12
Response to stimulus	0.0256	88
Animal organ development	0.0263	46
Regulation of cell adhesion	0.0278	18
Cellular response to chemical stimulus	0.0352	39
Tube development	0.0369	19
Establishment of skin barrier	0.0376	4
Positive regulation of cell migration	0.0398	14
Regulation of epithelial cell migration	0.0407	9
Regulation of multicellular organismal process	0.0454	40

Supplementary Table S5. Identified enriched functions and processes through overrepresentation analysis for upregulated differentially expressed proteins in comparison of patients with COPD and emphysema to healthy never-smokers.

Function description	FDR	Number of genes
Growth factor binding	0.00022	9
Calcium ion binding	0.00075	17
Thioredoxin peroxidase activity	0.0278	3
Fibronectin binding	0.0286	4
Insulin-like growth factor I binding	0.0286	3
Insulin-like growth factor II binding	0.0286	3
Laminin binding	0.0286	4
Structural constituent of skin epidermis	0.0356	4
Catalytic activity	0.0412	48
Hydrolase activity	0.0412	27
Cargo receptor activity	0.0412	5
Signaling receptor binding	0.0477	20
Process description	FDR	Number of genes
Response to chemical	0.00016	47
Epithelium development	0.00016	23
Tissue development	0.00039	28
Cellular response to chemical stimulus	0.00048	35
Epithelial cell differentiation	0.00069	16
Response to external stimulus	0.0034	31
Regulation of water loss via skin	0.0034	5
Response to stimulus	0.0034	66
Positive regulation of epithelial cell migration	0.0035	8

Response to organic substance	0.0039	33
Biological process involved in interspecies interaction between organisms	0.0049	23
Regulation of multicellular organismal process	0.0052	33
Immune system process	0.0057	28
Multicellular organismal process	0.0065	57
Response to other organism	0.0072	21
Anatomical structure development	0.01	48
Defense response	0.0117	21
Skin development	0.0124	9
Regulation of cell adhesion	0.0146	15
Animal organ development	0.0146	35
Response to oxygen-containing compound	0.0146	22
Establishment of skin barrier	0.0156	4
Digestion	0.0183	6
Positive regulation of endothelial cell migration	0.0183	6
Positive regulation of cell migration	0.0183	12
Regulation of cell migration	0.0194	16
Multicellular organismal homeostasis	0.0194	10
Positive regulation of multicellular organismal process	0.0214	21
Homeostatic process	0.0235	20
Cellular response to organic substance	0.0235	25
Epidermis development	0.0265	9
Immune response	0.029	19
Defense response to other organism	0.0296	16

Regulation of response to stimulus	0.0322	38
Positive regulation of endothelial cell chemotaxis	0.034	3
Epidermal cell differentiation	0.0433	7
Regulation of signal transduction	0.047	31

Supplementary Table S6. Identified enriched functions and processes through overrepresentation analysis for downregulated differentially expressed proteins in comparison of patients with COPD and emphysema to healthy never-smokers.

Function description	FDR	Number of genes
Structural constituent of chromatin	1.69e-23	18
Structural molecule activity	2.64e-17	25
Protein heterodimerization activity	1.42e-14	18
Protein dimerization activity	7.89e-08	19
Cadherin binding	2.34e-06	11
Cell adhesion molecule binding	3.59e-05	12
Antigen binding	5.05e-05	6
Protein tag	7.68e-05	4
Protein binding	0.00065	38
Structural constituent of skin epidermis	0.0025	4
DNA binding	0.0121	19
Nucleosomal DNA binding	0.0209	3
Epidermal growth factor receptor activity	0.0287	2
Process description	FDR	Number of genes
Nucleosome assembly	6.19e-18	16
DNA replication-dependent chromatin assembly	5.08e-14	10
Regulation of gene expression. epigenetic	3.76e-12	12
Telomere organization	5.75e-12	12
Protein-containing complex assembly	1.03e-05	18
Adaptive immune response	6.63e-05	10
Complement activation. classical pathway	0.0066	4
Histone H3-T3 phosphorylation involved in chromosome passenger complex localization to kinetochore	0.0376	2

Intermediate filament organization	0.0398	4
